# Tissue-resident and recruited mononuclear phagocyte organization in the cochlea under homeostasis and implant-induced inflammation

**DOI:** 10.3389/fncel.2026.1895870

**Published:** 2026-10-16

**Authors:** Shakila Mahmuda Fatima, Muhammad Taifur Rahman, Brian J. Mostaert, Ibrahim Razu, Bryce Hunger, Shanta Afrin, Sharar Naiarin Haque, Nashwaan Ali Khan, Zhenshen Zhang, Alexander D. Claussen, Marlan R. Hansen

**Affiliations:** Department of Otolaryngology-Head and Neck Surgery, University of Iowa, Iowa City, IA, United States

**Keywords:** CD11c, dendritic cells, inner ear immunity, macrophages, microglia, mononuclear phagocytes, TMEM119

## Abstract

**Background:**

The cochlea contains a diverse population of immune cells that contribute to tissue homeostasis and to responses to injury. Among these, mononuclear phagocytes including tissue-resident macrophages, dendritic cells, and microglia-like cells play central roles in immune surveillance and inflammation. Cochlear implantation induces a robust foreign body response, and here we sought to map the spatial distribution of mononuclear phagocytes across distinct cochlear compartments in healthy cochleae and following implantation.

**Materials and methods:**

We used two reporter mice in this study: C57BL/6-Tmem119em2Gfng/J (TMEM119+/GFP as reporter for microglia), B6. FVB-1700016L21RikTg (Itgax-DTR/EGFP)57Lan/J (CD11c+/GFP as reporter for dendritic cells). To define the spatial organization of different types of mononuclear phagocytes in normal cochleae and after implantation, 30-μm mid-modiolar cochlear sections from these reporter mice were imaged using a confocal microscope. Immunolabeling with cell-specific and activation markers was used to further characterize the cellular distributions and responses.

**Results:**

We identify heterogeneous distribution of mononuclear phagocytes throughout the cochlea. CD11c-positive dendritic cells display a more restricted baseline distribution, residing primarily in Rosenthal’s canal and the spiral ligament; they selectively accumulate at the implant–tissue interface in the scala tympani after implantation and upregulate major histocompatibility complex class II expression, consistent with antigen-presenting phenotypes. In addition, expression of microglia-associated markers, including TMEM119 and P2Y12, identifies a microglia-like population within specific cochlear sub-regions that persists following implantation.

**Conclusion:**

Together, these findings reveal an immune landscape in the cochlea under both healthy and implant-associated inflammatory conditions. CD11c-positive dendritic cells appear to play a central role in the inflammatory response to cochlear implants, becoming activated and localizing to the interface with the biomaterial surface.

## 1 Introduction

Sensorineural hearing loss (SNHL) typically results from irreversible damage to the sensory hair cells, supporting cells, and auditory neurons of the cochlea and represents a major cause of disability worldwide (Haile et al., 2024; Agrawal et al., 2008). Genetic predisposition, aging, noise exposure, and ototoxic drugs are well established contributors to cochlear pathology (Okano et al., 2008; Rahman et al., 2023b). Historically, the cochlea was regarded as an immune privileged organ due to the blood-labyrinth barrier and the apparent absence of classical lymphatic drainage (Fujioka et al., 2014; Harris and Ryan, 1995). More recently, this concept has been modified with the identification of resident immune cells in the cochlea and growing evidence of active immune surveillance under steady-state and disease conditions (Okano et al., 2008; Ginhoux et al., 2021; Davies et al., 2013). Here, using two reporter mouse lines, we investigate the identity and spatial organization of mononuclear phagocytes in the mouse cochlea under both homeostatic and cochlear implant-induced inflammatory conditions.

Mononuclear phagocytes, a heterogenous group of single nucleated immune cells with capacity for phagocytosis, consist of tissue-resident macrophages, monocytes, and dendritic cells (Chow et al., 2011; Guilliams et al., 2014). Among these cell types, monocytes are present in circulating blood (Karlmark et al., 2012). Tissue macrophages are dispersed in all tissues with diverse forms and functions (Gordon and Plüddemann, 2017). Dendritic cells, with a stellate morphology with long processes, represent professional antigen presenting cells with unique capacity to induce naïve T cells and their differentiation to effector T cells (Steinman and Cohn, 1973; Waisman et al., 2017). Various types of tissue-derived dendritic cells have been identified in the blood, spleen, lymph nodes, skin, lung, liver, gut, and kidney where they function to maintain immune tolerance and control immune responses. Dendritic cell dysfunction contributes to autoimmunity, allergy, and cancer (Chen et al., 2016). In the nervous system, an additional group of mononuclear phagocytes, ‘microglia’, has been described based on unique cell surface morphology (Giulian et al., 1995; Epelman et al., 2014; Hoeffel and Ginhoux, 2015). Macrophages, dendritic cells, and microglia have different embryonic origins, express different molecular markers, and perform divergent functions (Jurga et al., 2020; Chistiakov et al., 2017; Zhao et al., 2018; Liu and Nussenzweig, 2010; Nayak et al., 2014).

Although there are differences in ontogeny, morphology, and function among macrophages, microglia, and dendritic cells, distinguishing them based on molecular markers is challenging as there is significant overlap in the molecular markers of these cell types. Moreover, the expression of molecular markers in mononuclear phagocytes changes depending on functional status. IBA1, CX3CR1, CD14, CD64, CD68, CD11b, CD16, CD163 are examples of non-specific markers of mononuclear phagocytes (Jurga et al., 2020; Chistiakov et al., 2017; Zhao et al., 2018; Liu and Nussenzweig, 2010; Nayak et al., 2014). Currently, TMEM119 and P2Y12R are considered the two most specific markers of microglia (Jurga et al., 2020). CD11c appears to be the most specific marker for dendritic cells (Merad et al., 2013).

Hirose et al. reported that ‘mononuclear phagocytes’ exist in the mouse cochlea based on CD45 expression, a marker of nucleated hematopoietic cells, and morphological characteristics and that their number increases following noise exposure (Hirose et al., 2005). Later studies using CX3CR1-GFP reporter mice confirmed the presence of ‘mononuclear phagocytes’ in the cochlea (Sato et al., 2008; Sato et al., 2010; Sautter et al., 2006). Most commonly, mononuclear phagocytes have been described as ‘macrophages’. ‘Macrophage’ infiltration into the cochlea has been described following noise exposure (Frye et al., 2019), deafening (Kaur et al., 2015; Rahman et al., 2023a), cochlear implantation (Claussen et al., 2022; Noonan et al., 2020; Rahman et al., 2022), and age-related hearing loss (Seicol et al., 2022). Microglia have been identified in an *in vitro* neonatal rat cochlea model using the microglia-specific marker TMEM-119 (Seigel et al., 2017). However, the spatial organization of microglial cells in the adult cochlea remains poorly described (Chen et al., 2018; Faustmann et al., 2022). Meanwhile, dendritic cells have not been characterized in the cochlea.

A role of cochlear mononuclear phagocytes in adaptive immune responses in the cochlea is beginning to emerge. Mononuclear phagocytes expressing MHCII are present in the human cochlea and are associated with other cells involved in adaptive immunity (T and B lymphocytes) (Liu et al., 2021; Neefjes et al., 2011; Vivier and Malissen, 2005; Kawai and Akira, 2010; Mahla et al., 2013). Further, major histocompatibility complex II (MHCII) and class II transactivator (CIITA), two MHCII production cofactors, are expressed on mononuclear phagocytes (termed macrophages in the article) and their expression is up-regulated following acoustic trauma (Yang et al., 2015). Moreover, the basal portion of the cochlea, which demonstrates a predisposition to increased sensory cell loss following acoustic trauma, is spatially associated with the region of antigen-presenting activity. T and B lymphocytes infiltrate the cochleae following loud noise exposure (Rai et al., 2020; Nadol Jr et al., 2014). Likewise, B and T lymphocytes, foreign body giant cells, and macrophages all participate in an active cellular immune response triggered by the placement of an electrode array in the cochlea (Rahman et al., 2025). Together, these findings suggest that noise trauma and cochlear implantation activate an adaptive immune response in the cochlea.

The objective of this study is to evaluate the spatial organization of the two mononuclear phagocyte types (CD11c-positive dendritic cells, and TMEM119-positive tissue resident microglia-like cells) in the native cochlea and after cochlear implantation. We also evaluate MHCII expression on these cells to determine whether they are poised to mediate antigen presentation and, therefore, adaptive immune responses. The results provide insight into the role individual cell types may play in cochlear homeostasis and pathology, including innate and adaptive immune responses.

## 2 Methods and materials

### 2.1 Animals

The Institutional Animal Care and Use Committee (IACUC) of the University of Iowa approved all the animals used in this study. In this study, we used 2 different mouse lines:

1. C57BL/6-*Tmem119em2Gfng*/J (JAX stock #031823). This line is commonly called the TMEM119-2A-EGFP mouse. Transmembrane protein 119 (TMEM119) is a microglia-specific marker useful for discriminating the resident microglial population of the central nervous system from infiltrating immune cells (e.g., blood-derived macrophages). As a result of insertion of TMEM119-2A-EGFP knock-in allele with an enhanced green fluorescent protein gene (EGFP) into the stop codon of the transmembrane protein 119 locus (*TMEM119*), microglial cells express EGFP while retaining endogenous *TMEM119* expression (Kaiser and Feng, 2019). We have used homozygous female TMEM119-2A-EGFP (TMEM-119GFP/GFP) mice of C57BL/6 J background to breed with C57BL/6 J male mice (JAX stock #000664). The heterozygous (TMEM-119GFP/+) offspring mice were used as microglia-specific reporter mice.

2. B6. FVB-*1700016L21RikTg(Itgax-DTR/EGFP)57Lan*/J(JAX stock #004509). This line is commonly called the CD11c-DTR mouse or CD11c-DTR/GFP transgenic mouse. Under the direction of the CD11c promoter (*Itgax*), a diphtheria toxin receptor - enhanced green fluorescent protein (DTR/EGFP) fusion protein is expressed in dendritic cells of these mice. Mice with these transgenes are depleted of dendritic cells upon diphtheria toxin (DT) administration (Jung et al., 2002). CD11c-DTR/GFP transgenic mice function as useful reporter mice for dendritic cells and to determine the specific role of dendritic cells in the immune response. For our study, we used female heterozygotes of CD11c-DTR/GFP mice (CD11cGFP/+) of C57BL/6 J background, bred them with C57BL/6 J male mice (JAX stock #000664). Following genotyping, heterozygous offspring (CD11cGFP/+) were used as dendritic cell reporter mice in our study.

Male and female mice from both mouse models were euthanized at 10 -12 weeks of age for use in this study. In total, 7 CD11c-DTR/GFP mice were used: 3 for baseline (native) regional density mapping and 4 for the paired implanted-versus-contralateral comparison. Similarly, 6 TMEM119-2A-EGFP mice were used: 3 for baseline mapping and 3 for the paired implanted-versus-contralateral comparison. Additional mice of each line were used for reporter validation (diphtheria toxin-mediated depletion and brain co-localization studies, respectively).

**Figure 1:**
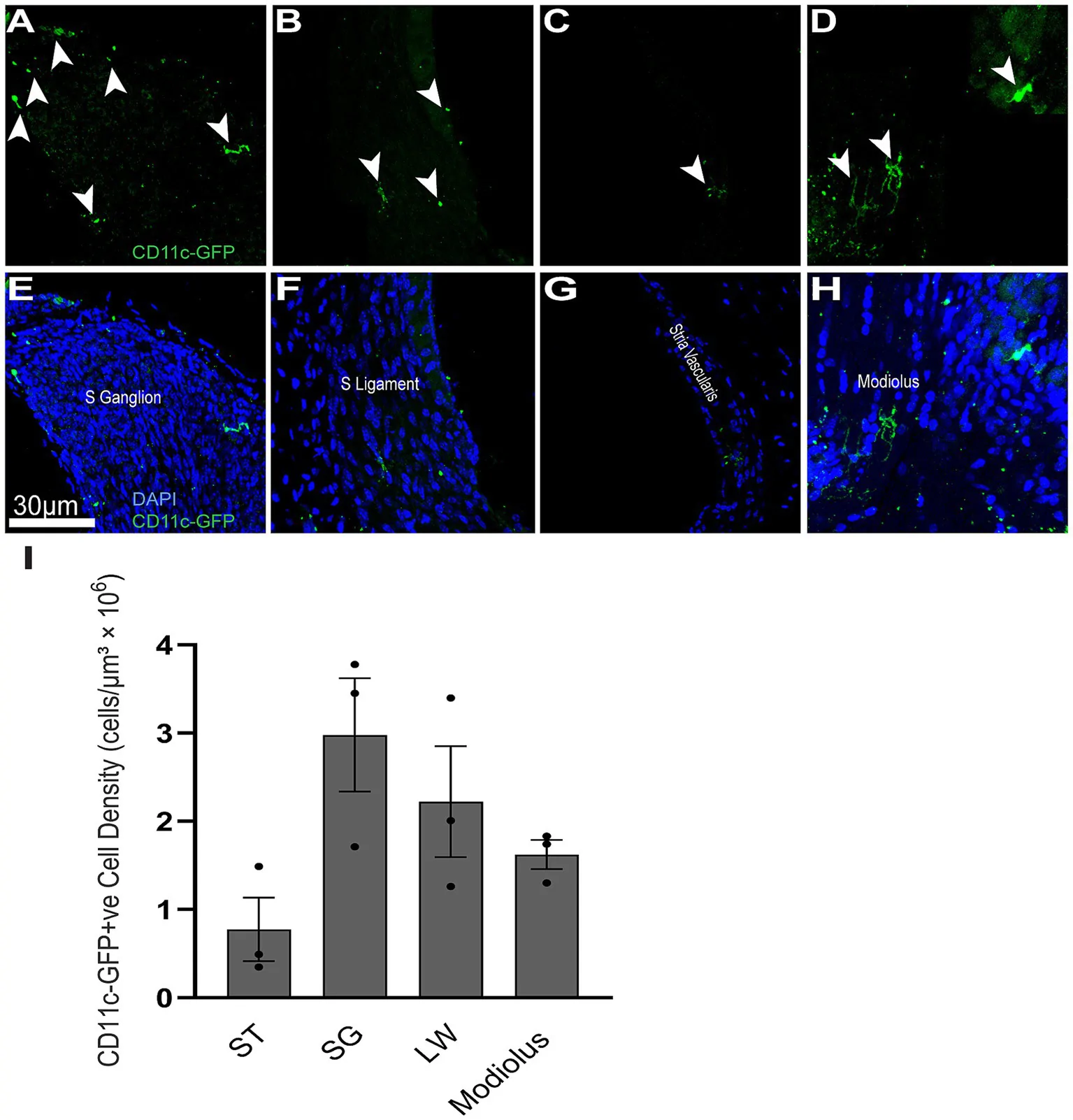
Spatial distribution of CD11c-GFP^+^ cells in the healthy cochlea. **(A–D)** CD11c-GFP^+^ cells (green) in four cochlear regions, with arrowheads indicating positive cells. **(E–H)** Corresponding merged images with DAPI (blue), showing anatomical landmarks: spiral ganglion **(E)**, spiral ligament **(F)**, stria vascularis **(G)**, and modiolus **(H)**. Scale bar = 30 μm. **(I)** Quantification of CD11c-GFP^+^ cell density (cells/μm^3^ × 10^6^) across four cochlear regions of the basal turn; scala tympani (ST), spiral ganglion (SG), lateral wall (LW), and modiolus. Data are mean ± SEM; (*n* = 3 per region).

**Figure 2:**
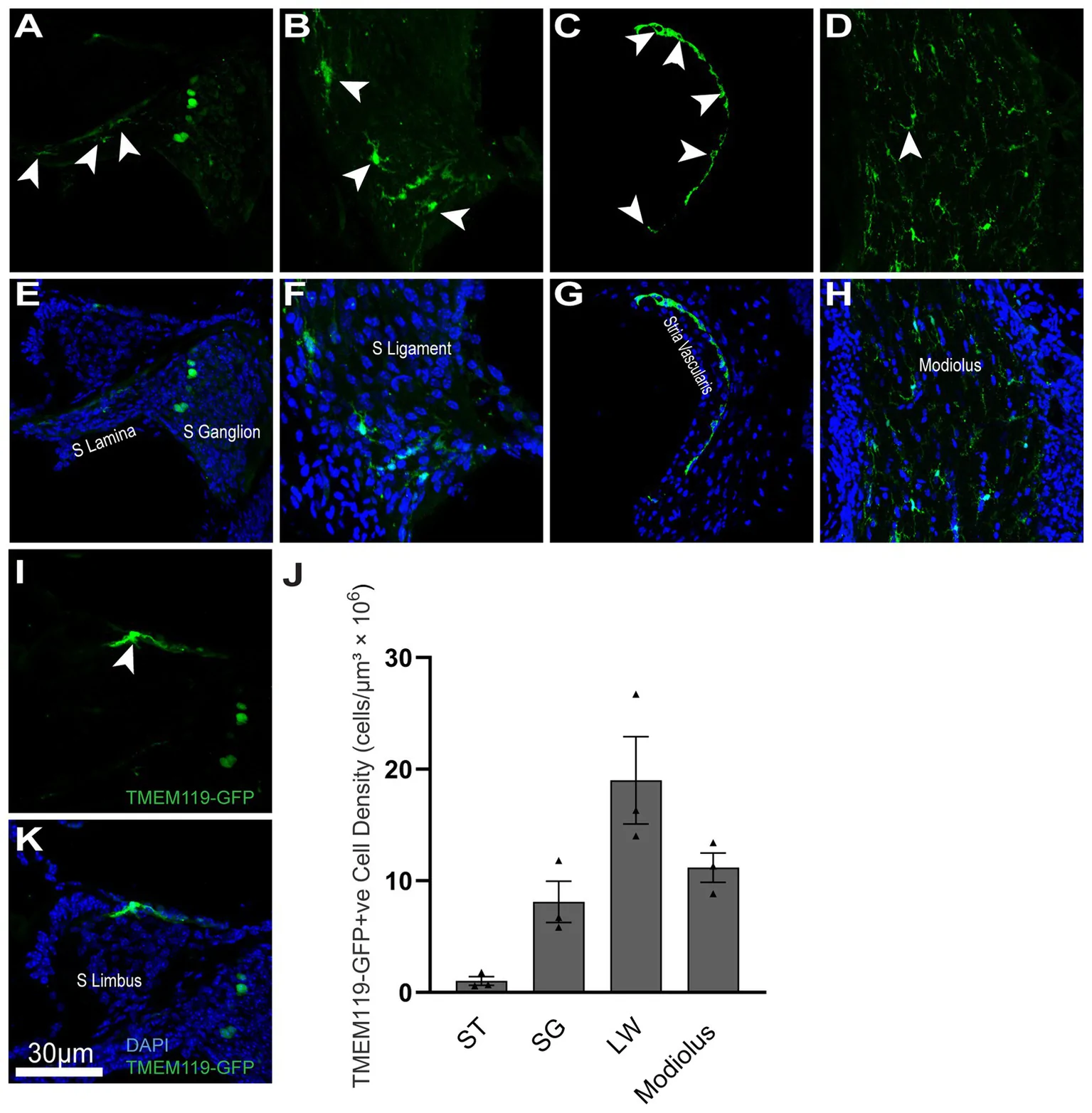
Spatial distribution of TMEM119-GFP^+^ cells in the healthy cochlea. **(A–D)** TMEM119-GFP^+^ cells (green) with arrowheads indicating positive cells. **(E–H)** Corresponding merged images with DAPI (blue), showing tissue architecture of spiral ganglion and spiral lamina **(E)**, spiral ligament **(F)**, stria vascularis **(G)**, and modiolus **(H)**. (I, K) TMEM119-GFP^+^ cells **(I)**, and with DAPI (blue, **K**), at the spiral limbus. Scale bar = 30 μm. **(J)** Quantification of TMEM119-GFP^+^ cell density (cells/μm^3^ × 10^6^) across four cochlear regions of the basal turn; scala tympani (ST), spiral ganglion (SG), lateral wall (LW), and modiolus. Data are mean ± SEM; (n = 3 per region).

**Figure 3:**
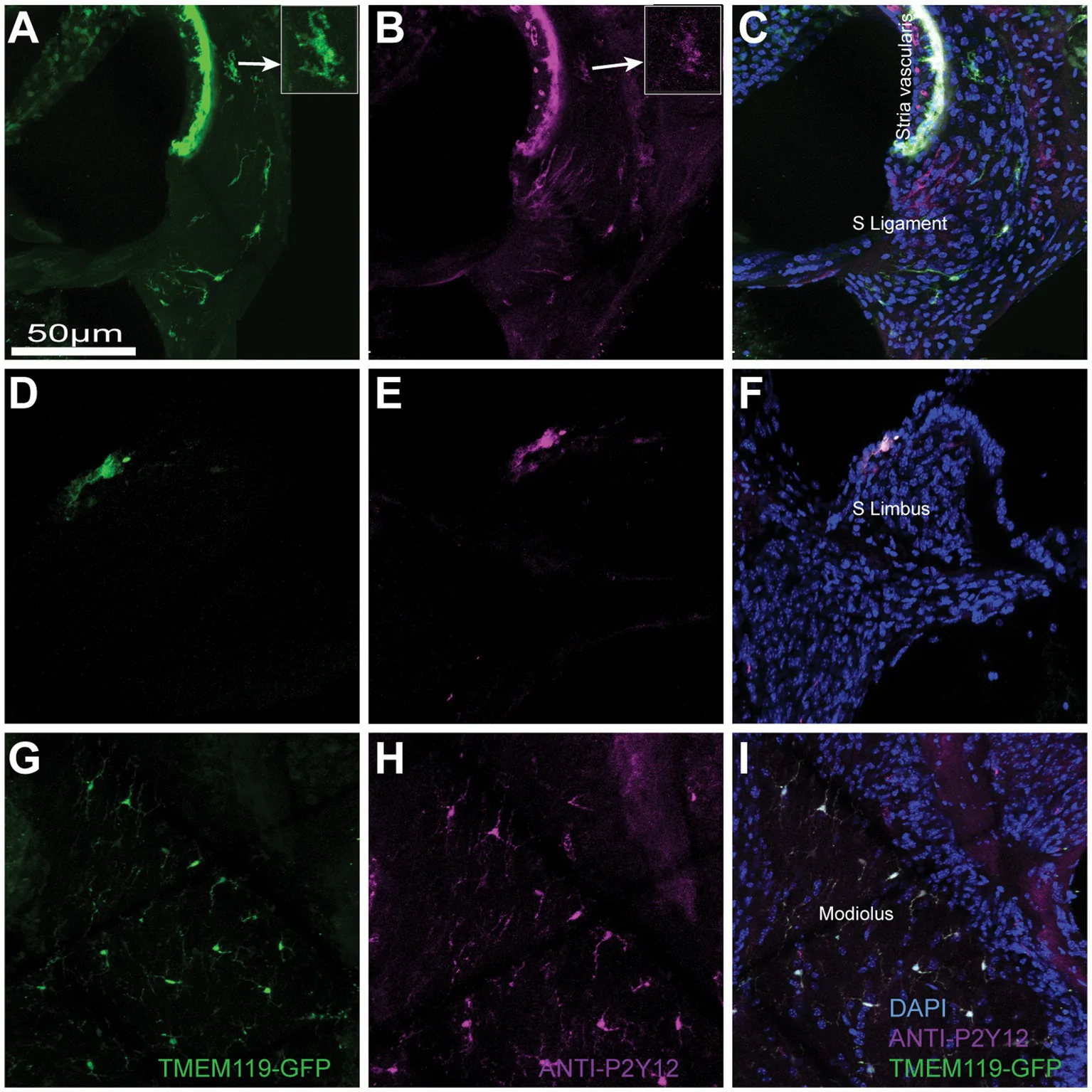
Co-localization of TMEM119-GFP and anti-P2Y12 in cochlear compartments. **(A–D)** Representative confocal images of spiral ligament and stria vascularis showing TMEM119-GFP^+^ cells (green), P2RY12 signal (magenta), and merged channels with DAPI nuclear counterstain (blue). Arrows indicate cells of interest. Insets show enlarged views of the boxed regions from the same fields. **(D–F)** Corresponding single-channel views highlighting TMEM119-GFP^+^ cells (green), anti-P2RY12 (magenta), and DAPI (blue) distribution in spiral limbus. **(G–I)** Representative TMEM119-GFP^+^ cells and P2Y12 signal in higher-density region, modiolus. Scale bar = 50 μm.

**Figure 4:**
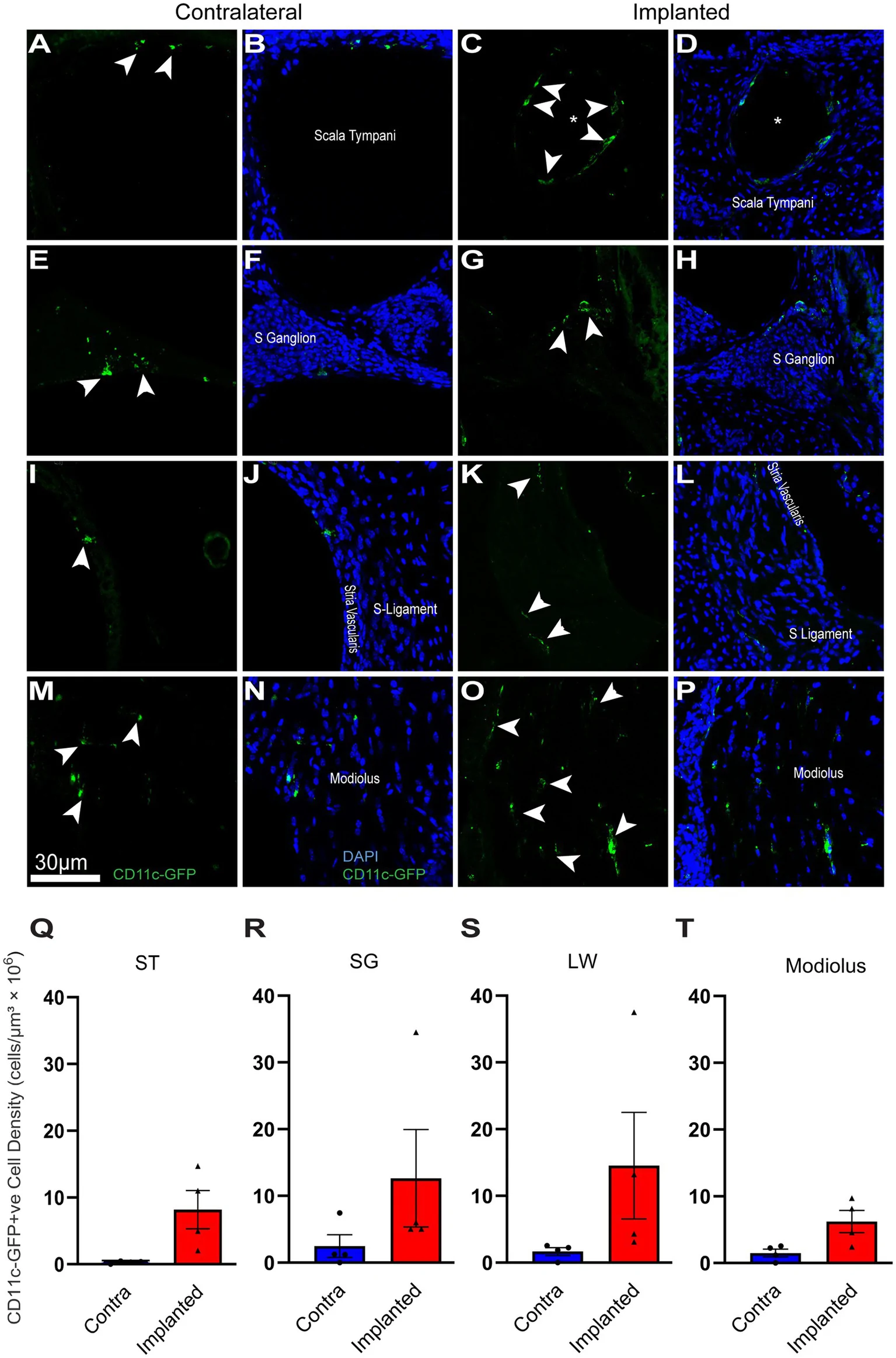
CD11c-GFP^+^ cells distribution and quantification of density in the implanted cochlea relative to the contralateral side. **(A–T)** CD11c-GFP^+^ cells (green) and DAPI (blue) in contralateral (non-implanted; left columns) and implanted (right columns) cochleae across four regions: scala tympani **(A–D)**, spiral ganglion **(E–H)**, spiral ligament & stria vascularis **(I–L)**, and modiolus **(M–P)**. Arrowheads indicate CD11c-GFP^+^ cells; asterisks (*) mark the implant tract. Scale bar = 30 μm. **(Q–T)** Quantification of CD11c-GFP+ cell density (cells/μm3 × 106) in the basal turn, comparing contralateral (blue) and implanted (red) cochleae across four regions, scala tympani (ST, **Q**), spiral ganglion (SG, **R**), lateral wall (LW, **S**), and modiolus **(T)**. Data represent mean ± SEM (*n* = 4 paired samples per region); paired t-tests were used for statistical comparisons (*p < 0.05).

### 2.2 Genotyping

Genotyping was done for C57BL/6-*Tmem119em2Gfng*/J, B6.FVB-*1700016L21RikTg(Itgax-DTR/EGFP)57Lan*/J using standard PCR of genomic DNA from tail samples. The vendors suggested the PCR probes, which are available from Jackson Laboratories.

### 2.3 Cochlear implantation

We performed cochlear implantation using an established murine model, as described in prior studies (Rahman et al., 2023a, b; Claussen et al., 2022; Noonan et al., 2020; Rahman et al., 2022; Rahman et al., 2025). In brief; we used young adult mice (10–12 weeks of age) and anesthetized them with ketamine (80 mg/kg) and xylazine (10 mg/kg), then placed on a heated surgical platform. A postauricular approach exposed the temporal bone, and the otic bulla was opened to visualize the cochlea. A small cochleostomy was made in the basal turn of the scala tympani. A custom electrode array was inserted into the scala tympani through the cochleostomy to set depth. The electrode array was secured, and the site was closed in layers. Mice recovered under standard postoperative monitoring until fully alert. Animals with implants were observed for 28 days post-implantation (DPI) to represent the chronic phase of the foreign body response (Noonan et al., 2020; Rahman et al., 2022), before tissue harvest and analysis. All procedures complied with protocols approved by the institutional animal care and use committee (IACUC) and followed relevant guidelines for laboratory animal care.

### 2.4 Diphtheria toxin–mediated depletion of CD11c^+^ cells

CD11c-DTR/GFP mice were treated with diphtheria toxin to deplete CD11c^+^ cells. Diphtheria toxin was diluted in sterile PBS and administered intraperitoneally at 8 ng/g body weight. Tissue was collected approximately 24 h after injection for analysis of CD11c-GFP. Gut tissue without toxin, treated with only PBS was used as a positive control for CD11c-GFP, while diphtheria toxin–treated CD11c-DTR/GFP positive gut tissue served as the negative/depletion control (Jung et al., 2002).

### 2.5 Immunohistochemistry

Ketamine (80 mg/kg) and xylazine (10 mg/kg) were used to anesthetize 10–12 weeks old mice prior to transcardial perfusion with ice-cold phosphate-buffered saline (PBS), followed by 4% paraformaldehyde (PFA). Cochleae were collected from both reporter strains. After harvesting cochleae, they were fixed overnight with 4% paraformaldehyde at 4 °C in the dark. Excess PFA was removed by rinsing the samples with PBS overnight in a rotator. Cochleae were decalcified in 0.12 M EDTA (pH 7.4) solution on a rotator; EDTA was changed daily for 3–5 days. Following decalcification, cochleae were rinsed with PBS for 3 times, 10 min each wash. Cryoprotection of decalcified cochleae was performed using a series of sucrose solutions, starting at 10% and increasing by 10% every hour until reaching 30% at the end. Until sectioned, cryoprotected mice cochleae were stored at −20 °C. O. C. T. embedding medium (Tissue-TEK) was infused into the cochlea, and the sample was mounted on the stage of a sliding block microtome (American Optical 860) with O. C. T. and dry ice. Mounted cochleae were sectioned at 30 μm parallel to the mid-modiolar plane. Until immunolabeling or nuclear labeling was performed, sections on Fisher Superfrost slides were stored at –20 °C.

To determine the spatial organization of cell types (microglia-like cells, and dendritic cells), during immunolabeling, slides were first warmed to room temperature (~20–22 °C). Then, using a Kapton Jar, slides were washed (3 × 5 min each wash) with ‘washing buffer’ containing 0.1% Triton X-100 and 0.1% Tween-20 in Tris-Buffered Saline (TBS). Permeabilization and blocking were done simultaneously in a ‘blocking buffer’ for 2 h. ‘Blocking buffer’ contained 0.1% Triton X-100 and 0.1% Tween-20, 1% bovine serum albumin (RPI, CAS#9048-46-8) and 0.02% sodium azide (Sigma, catalog #S2002) in TBS. After blocking, sections on slides were incubated with the primary antibody. The following primary antibodies were used in this study, anti-P2Y12 is a rat monoclonal antibody, basically, a rat IgG2b from Biolegend (Catalog#848001, RRID: AB_2650633; used at 1:200) and MHC class II (I-A/I-E) monoclonal antibody (M5/114.15.2), a rat IgG2b antibody, eBioscienceTM (Catalog # 14-5321-82, RRID: AB_467561; used at 1:200). The primary antibody in blocking buffer was applied at day 1, followed by washing of the sections (3 × 5 min) in ‘washing buffer’ at day 2. Samples were then incubated in IgG goat anti-secondary antibodies (Alexa Fluor conjugates, 1:400) for 2 h at room temperature. Following a wash (3 × 5 min) with ‘washing buffer’, Nuclei were stained by incubating the samples in Hoechst 33342 (10 μg/mL in TBS, Sigma) for 15 min at room temperature. Following wash with ‘1X TBS (Tris Buffer)’ (3 × 5 min), samples were cover-slipped with Aqua Mount mounting medium (Tris Buffer catalog #17985–10, Electron Microscopy Sciences).

Brain samples were collected from TMEM119-GFP mice, fixed overnight in 4% paraformaldehyde, cryopreserved, embedded in O. C. T., and 45 μm-thick sections parallel to the coronal plane were labeled with Hoechst 33342, and immunofluorescences and imaged. Small intestine samples were collected from CD11c-DTR/GFP mice, fixed overnight with 4% paraformaldehyde, cryopreserved, and embedded in O. C. T. 30 μm thick sections perpendicular to the axis of the small intestine were labeled with Hoechst 33342, and imaged.

### 2.6 Imaging

For histological analysis of nuclei, microglia like cells, and dendritic cells, 30 μm thick sections from the implanted, contralateral, and uninjured healthy cochlea were immunolabeled, as described above, to visualize different types of mononuclear phagocytes (EGFP) and immunolabeled markers (P2Y12 and MHCII). Fluorescently labeled cochlear sections (2 per cochlea) were imaged at a magnification of 40x (1.20 NA), 1x digital zoom, z-axis spacing of 0.79 μm, and constant exposure/gain settings throughout the experiment using a Leica Stellaris 5 confocal system. Maximum-intensity z-projections of 3D confocal image stacks were generated using IMARIS image analysis software. The adjustment of intensity and gain of Rosenthal’s canal, stria vascularis, spiral ligament, margin of the scala tympani, and modiolus was done in the IMARIS image analysis software. In the implanted cochlea, imaging focused on both areas along the electrode array trajectory and on anatomically matched regions outside the implant. Imaging was completed in a single session to limit variability between samples. Images were saved in a lossless format.

To confirm reporter specificity and provide reference morphology, control tissues were imaged under the same conditions. Small intestine from CD11c-DTR/GFP mice and brain samples from TMEM119-GFP mice were imaged too, using a 40X objective (0.70NA) with a z-axis spacing of 0.79 μm. We did not perform any nonlinear image adjustments to individual images. However, any linear adjustments were applied uniformly across all samples.

### 2.7 Image analysis

We prepared the images in IMARIS (Oxford Instruments) image analysis software. To examine the spatial distribution, morphology, and anatomical localization of mononuclear phagocytes across cochlear subregions, we used Z-stack images and maximum-intensity projections. We defined regions of interest based on cochlear anatomy using nuclear labeling alone in order to avoid bias from reporter or immunolabeling channels. Identification of mononuclear phagocytes was based on reporter expression. Tissue resident microglia-like cells were identified with TMEM119-GFP and dendritic cells by CD11c-GFP expression.

Analysis focused on describing the location of these cells, their distribution across cochlear subregions, density of these cells and how their morphology differed between implanted and unimplanted conditions. We processed all imaging steps consistently across samples. Fluorescently labeled cochlear sections (two per cochlea) were imaged as described above using a Leica Stellaris 5 confocal system. Maximum intensity z-projections of 3D confocal image stacks were analyzed with IMARIS image analysis software to quantify cell counts and volumes of segmented cochlear regions of interest. The outline of Rosenthal’s canal, lateral wall, and margin of the scala tympani was drawn in the basal turns of the cochlea. Additionally, the modiolus of the cochleae was segmented as a separate entity. Volumetric measurement of each area was done. Tissue resident microglia (TMEM119^+/GFP^) and dendritic cells (CD11c^+/EGFP^) were counted in each of the 4 areas and the density of the cells in each area was quantified. Values from 2 sections per animal were averaged to determine individual subject values, thus “n” references the total number of mice used in the study. Images were captured in the same session using identical settings for imaging.

### 2.8 Statistical analysis

Statistical analyses were performed using GraphPad Prism 11. For healthy, uninjured cochleae, we report density of CD11c-GFP^+^ and TMEM119-GFP^+^ populations as mean values for each of four anatomical regions of the basal turn, scala tympani (ST), spiral ganglion (SG), lateral wall (LW), and modiolus to describe baseline regional distribution. Cell density data for CD11c-GFP^+^, and TMEM119-GFP^+^ populations were also compared between contralateral and implanted cochleae across these same four anatomical regions in basal turn, scala tympani (ST), spiral ganglion (SG), lateral wall (LW), and modiolus using paired t-test. Data are presented as mean values, and statistical significance was defined as *p* < 0.05.

## 3 Results

### 3.1 Spatial organization of mononuclear phagocytes in the uninjured cochlea

To define the baseline immune landscape of the cochlea, we first characterized the distribution of mononuclear phagocytes in healthy adult mouse cochleae. We used reporter-based and immunohistochemical approaches. We observed that cochlear sections revealed multiple mononuclear phagocyte populations distributed across distinct anatomical compartments. Figures 1, 2 show the widespread distribution of CD11c-GFP^+^ dendritic cells and TMEM119-GFP^+^ microglia like cells in healthy adult (10–12 weeks old) mouse cochlea. CD11c-GFP positive cells were consistently identified within sub-regions of Rosenthal’s canal (Figure 1A), particularly in proximity to spiral ganglion neuron cell bodies. In 30 μm-thick sections, these CD11c-GFP-positive cells displayed small punctate to stellate profiles and were scattered, individually interspersed among neuronal and glial elements rather than dense cluster. Validation of CD11c–GFP reporter expression is shown in Supplementary Figure S1.

We also observed dendritic cells within the spiral ligament and stria vascularis (Figures 1B,C), indicating that the CD11c-GFP-positive cells were associated with connective tissue and vascular structures. The Rosenthal’s canal consistently emerged as a hub of dendritic cell localization, distinct from the macrophage-rich regions of the lateral wall and modiolus (Hirose et al., 2005). These findings indicate that, under steady-state conditions, CD11c-GFP^+^ dendritic cells displayed regional heterogeneity (Figure 1), with the spiral ganglion (SG) having the highest mean density (~2.9 × 10^6^ cells/μm^3^), followed by the lateral wall (LW; ~2.0 × 10^6^ cells/μm^3^) and modiolus (~1.3 × 10^6^ cells/μm^3^). The scala tympani (ST) showed the lowest density among the regions examined (~0.7 × 10^6^ cells/μm^3^). We examined the density of these four regions of basal turn. These findings suggest that CD11c-GFP^+^ mononuclear phagocytes are not uniformly distributed throughout the cochlea but instead show region-specific patterns of density consistent with distinct local microenvironments across cochlear compartments (Figure 1).

Figure 2 shows that resident microglia-associated markers were also detected in the uninjured, healthy cochlea (10–12 weeks old) under steady-state conditions. Before using these markers, we validated them in the brain (Supplementary Figure S2). TMEM119-GFP reporter expression revealed a population of cells with resident microglia-like molecular signatures distributed within specific cochlear regions, including stria vascularis (Figures 2C,G) near the blood-labyrinth barrier, modiolus (D, H), spiral ligament (B, F), spiral limbus (I, K), and bony spiral lamina (A, E). Complementary immunolabeling for P2Y12 (100 μm thick sections, used specifically for this qualitative co-localization panel to maximize antibody penetration and signal intensity; full z-stacks were displayed as maximum-intensity projections to ensure uniform labeling was captured throughout section depth) further identified cells with microglia-associated phenotypes in overlapping regions in healthy, uninjured cochlea (Figures 3B,E,H). This panel is not used for the quantitative density comparisons reported elsewhere in this study, which were all performed on matched 30 μm sections.

**Figure 5:**
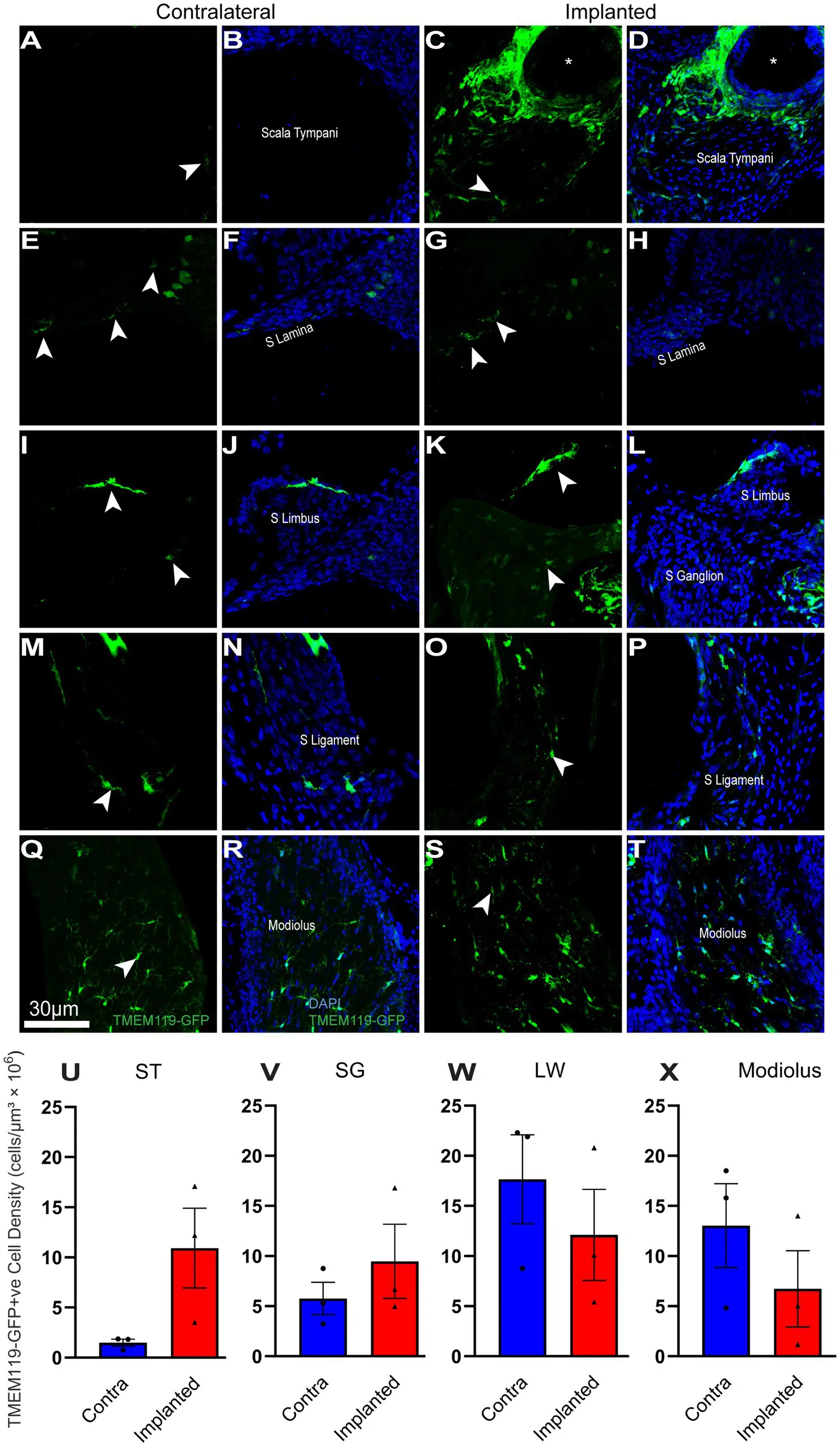
TMEM119-GFP^+^ cell density in the implanted cochlea relative to the contralateral side. **(A–C)** TMEM119-GFP+ cells (green) and DAPI (blue) in contralateral (non-implanted; left columns) and implanted (right columns) cochleae, shown across the scala tympani **(A–D)**, spiral lamina **(E–H)**, spiral limbus and spiral ganglion **(I–L)**, spiral ligament **(M–P)**, and modiolus **(Q–T)**. Arrowheads indicate TMEM119-GFP^+^ cells; asterisks (*) mark the implant tract. Scale bar = 30 μm. **(U–X)** Quantification of TMEM119-GFP^+^ cell density (cells/μm3 × 106) in contralateral (blue) versus implanted (red) cochleae for scala tympani (ST, **U**), spiral ganglion (SG, **V**), lateral wall (LW, **W**), and modiolus **(X)**. Data are mean ± SEM; n = 3 paired samples per region. Statistical comparisons were performed using paired t-tests; none of the pairwise comparisons reached statistical significance (*p* > 0.05).

We examined TMEM119-GFP^+^ cell density across cochlear regions of basal turn in the healthy cochlea (Figure 2J). TMEM119-GFP^+^ cells were detectable in all regions examined, with the lateral wall (LW) showing the highest mean density (~17.8 × 10^6^ cells/μm^3^), followed by the modiolus (~9.3 × 10^6^ cells/μm^3^) and spiral ganglion (SG; ~7.1 × 10^6^ cells/μm^3^). The tissues lining the scala tympani (ST) showed minimal TMEM119-GFP^+^ cell density (~0.5 × 10^6^ cells/μm^3^). In addition to these four quantified regions, TMEM119-GFP^+^ cells were consistently observed at the spiral limbus (Figures 2I,K).

### 3.2 Mononuclear phagocytes accumulation and persistence following cochlear implantation

#### 3.2.1 Dendritic cell recruitment and distribution, and activation at the implant interface

Following cochlear implantation we see a dynamic redistribution of dendritic cells in the cochlea (Figure 4). CD11c–GFP positive cells were prominently detected at the implant-tissue interface at 28 DPI; at this interface, these cells are located in close proximity to the electrode array surface. In the scala tympani, the number of dendritic cells increased following implantation in comparison to contralateral (Figures 4A–D), indicating their recruitment or local expansion in response to surgical trauma and biomaterial exposure.

We compared cell density between the implanted and contralateral (non-implanted) cochlea within 4 regions of the base (scala tympani, spiral ganglion, lateral wall and modiolus) using paired t-tests (Figures 4Q–T). CD11c-GFP^+^ cell density trended higher in the implanted cochlea relative to the contralateral side across all four regions examined. This increase reached statistical significance in the modiolus (paired t-test, t = 4.153, df = 3, *p* = 0.0254; mean difference = 4.718 ± 2.272 cells/μm^3^ × 10^6^, 95% CI: 1.102 to 8.333). Differences did not reach statistical significance in the scala tympani (ST; t = 2.772, df = 3, *p* = 0.0695), spiral ganglion (SG; t = 1.287, df = 3, *p* = 0.2885), or lateral wall (LW; t = 1.639, df = 3, *p* = 0.1997), although a trend toward increased density in the implanted cochlea was observed for each of these regions as well.

In Supplementary Figure S3, we observe a defining feature of dendritic cell activation following implantation: upregulation of major histocompatibility complex class II (MHCII). At 28 DPI, there was strong MHCII expression on CD11c-GFP-positive cells at the implant interface.

#### 3.2.2 Identification of microglia-like cells in the implanted cochlea

Figure 5 represents the analysis of TMEM119- GFP reporter and demonstrates the presence of microglia-like cells in the cochlea following implantation at 28 DPI. At 28 DPI, TMEM119-GFP-positive cells were detected in the scala tympani, spiral limbus, spiral ligament, bony spiral lamina, and modiolus. Compared with the contralateral (Figures 5M,N) and healthy (Figures 2C,G) cochlea, TMEM119-GFP expression was reduced in the stria vascularis following implantation (Figures 5O,P). To determine whether cochlear implantation altered resident TMEM119-GFP^+^ cell density, we compared TMEM119-GFP^+^ cell density between the implanted and contralateral (non-implanted) cochlea within each region using paired t-tests (Figures 5U–X). Unlike the increase observed for CD11c-GFP^+^ cells following implantation (Figure 4), TMEM119-GFP^+^ cell density did not change significantly with implantation in any of the four regions examined. In the scala tympani, density trended higher (Figure 4U) in the implanted cochlea (paired t-test, t = 2.596, df = 2, *p* = 0.1219; mean difference = 9.451 ± 6.306 cells/μm^3^ × 10^6^, 95% CI: −6.213 to 25.11), as did (Figure 5V) the spiral ganglion (SG; t = 1.712, df = 2, *p* = 0.2291; mean difference = 3.710 ± 3.754, 95% CI: −5.615 to 13.04). In contrast, density trended lower (Figure 5W) in the implanted cochlea in the lateral wall including stria vascularis (LW; t = 1.638, df = 2, *p* = 0.2430; mean difference = −5.550 ± 5.868, 95% CI: −20.13 to 9.027) and the modiolus (Figure 5X) (t = 2.812, df = 2, *p* = 0.1066; mean difference = −6.303 ± 3.883, 95% CI: −15.95 to 3.342). None of these differences reached statistical significance (*n* = 3 paired samples per region).

## 4 Discussion

### 4.1 Distinct mononuclear phagocyte populations populate the adult healthy cochlea

Since Ilya Metchinkoff (1892) described the phagocyte system, knowledge of mononuclear phagocyte cells and their function has expanded significantly, as recently reviewed by Guilliams et al. (2014). The classification of these cell populations has evolved through phases. The term’ mononuclear phagocyte system’ was coined in 1972 with two members of the family: monocyte and macrophage (van Furth et al., 1972). The underlying assumption of this system was that all macrophages come from blood monocytes. Soon after the discovery of ‘dendritic cells’ in 1973, these cells were incorporated into the nomenclature (van Furth, 1981; Kassianos et al., 2015). Since then, the mononuclear phagocytes have been classified according to these categories in most organs. In the cochlea, however, the classification of mononuclear phagocytes has not been fully characterized. This study provides a comprehensive spatial and phenotypic characterization of two mononuclear phagocytes, CD11c^+^ dendritic cells and TMEM119^+^ microglia-like cells in the mammalian cochlea under homeostatic conditions and after cochlear implantation, a clinically relevant model of biomaterial-induced neuroinflammation (Foggia et al., 2019). We integrated reporter-based lineage tracing with immunophenotypic validation to demonstrate that the cochlea harbors multiple, distinct mononuclear phagocyte populations, including dendritic cells (CD11c-GFP^+^), and tissue resident microglia-like cells (TMEM119-GFP^+^) (Butovsky et al., 2014; Bennett et al., 2016; Krasemann et al., 2017; Masuda et al., 2019; Haynes et al., 2006; Jordão et al., 2019; Prinz et al., 2021). Each of these cell types occupies specific anatomical niches and exhibits differential responses to implantation. These data advance the current understanding of cochlear immune surveillance and the position of dendritic cells (Yang et al., 2015; Rai et al., 2020; Nadol Jr et al., 2014; Jung et al., 2002)and microglia (Prinz et al., 2021), which have previously been underappreciated contributors to the cochlear foreign body response. These findings complement the established role of macrophages in fibrotic tissue remodeling and chronic inflammation (Rahman et al., 2023b; Foggia et al., 2019; Anderson et al., 2008).

In reference to the cochlea, the term ‘mononuclear phagocytes’ has often been replaced by ‘macrophages’ based on the expression of non-specific markers without further molecular characterization. In rare instances, cells morphologically reminiscent of dendritic cells, lacking the classic dendritic cell marker CD11c, have been described in cochleae (Yang et al., 2015; Rai et al., 2020; Nadol Jr et al., 2014; Jung et al., 2002). Microglia, which have a similar ontogeny to ‘tissue macrophages’, have been considered a cell limited to the central nervous system (CNS) (Prinz et al., 2021; Cederroth et al., 2019). Therefore, it is not surprising that microglia have not been heavily investigated in the cochlea, as it is considered part of the peripheral sensory nervous system. In one study, microglia have been reported in the rat cochlea, using a microglia-specific anti-TMEM119 antibody (Seigel et al., 2017). Gene expression profiling in the mouse cochlea also demonstrated the presence of gene transcripts for TMEM119 (gene for Transmembrane protein 119) and Itgax (gene for CD11c) in the gEAR database^1^, described by Cederroth et al. (Forrester et al., 2010). Considering the paucity and, to some extent, discrepancy of data from immunolabeling and gene expression profiling of microglia and dendritic cells, we sought to verify the presence or absence of different types of mononuclear phagocytes using a more reliable technique (transgenic fluorescent reporter mice).

Our findings expand the traditional view of cochlear immunity, in which most resident immune cells were broadly categorized as macrophages. Instead, the present data support a more complex immune architecture in which cell identity, location, and response to injury are linked. The compartment-specific distribution and the density comparisons shown in Figures 1, 2 suggest that immune surveillance in the cochlea is regionally structured rather than diffuse. This is similar to the mononuclear phagocyte system in the brain, where microglia, dendritic cells, resident macrophages, and monocyte-derived infiltrating macrophages have been documented (Okunuki et al., 2019). Among other special senses, the retina, which is considered part of the central nervous system, has a similar composition of mononuclear phagocytes, including dendritic cells (Yang et al., 1996), microglia (Batbold et al., 2017), and macrophages (Feng et al., 2023). However, unlike the central nervous system, microglia are few in number in the cochlea, whereas macrophages are relatively abundant. Macrophages, but not microglia, are observed in peripheral ganglia, including the dorsal root, trigeminal, and sympathetic ganglia (Lucinda et al., 2017; Oggero et al., 2022; Pirzgalska et al., 2017; Zhang et al., 2021). Further studies are warranted on other parts of the peripheral nervous system to investigate the types of mononuclear phagocytes using transgenic reporter animal models.

### 4.2 Dendritic cells may coordinate adaptive immune responses in the implanted cochlea

A major finding of this study is the identification of CD11c^+^ dendritic cells as a distinct cochlear immune cell population. In Figure 1 we described the spatial distribution of CD11c^+^ dendritic cells under steady-state conditions. CD11c-positive cells were localized primarily to Rosenthal’s canal, spiral ligament, stria vascularis, and modiolus, suggesting defined antigen-surveillance niches adjacent to neurons, vasculature, and connective tissue. The density distribution findings (Figure 1I) suggest that CD11c-GFP^+^ mononuclear phagocytes are not uniformly distributed throughout the cochlea, but instead show region-specific patterns of density consistent with distinct local microenvironments across cochlear compartments.

This restricted distribution contrasts with the broader macrophage network and indicates functional specialization (Li and Barres, 2018; Salt and Hirose, 2018). A similar distribution of dendritic cells has been reported in other sensory organs, including the cornea and meninges, where they function as immune sentinels capable of initiating adaptive immune responses (Steinman et al., 1999; Forrester and McMenamin, 2025).

Following implantation, dendritic cells redistributed toward the implant interface and showed increased density (Figure 4). This upward trend in CD11c-GFP^+^ cell density across all four regions examined (Figures 4Q–T), reaching statistical significance only in the modiolus (paired t-test, t = 4.153, df = 3, *p* = 0.0254; mean difference ≈ 4.72 × 10^6^ cells/μm^3^). The modiolus houses the spiral ganglion neuron cell bodies and lies in closest proximity to the electrode array trajectory, making it a plausible focal point for implant-induced tissue disruption, direct mechanical trauma, and subsequent recruitment of CD11c-GFP^+^ phagocytes. The trends toward increased density in the scala tympani base, spiral ganglion base, and lateral wall base, although not reaching statistical significance, are directionally consistent with a broader, implant-associated inflammatory response that may achieve statistical significance with a larger sample size.

Another important finding is that these CD11c^+^ dendritic cells strongly expressed MHCII (Supplementary Figure S3), consistent with acquisition of antigen-presenting function. This suggests that dendritic cells may link innate injury sensing with adaptive immune activation in the implanted cochlea.

The possibility of sustained antigen presentation has important implications. Cochlear implantation introduces permanent foreign material while simultaneously causing tissue disruption (Merad et al., 2013; Noonan et al., 2020; Rahman et al., 2022; Seyyedi and Nadol, 2014; O'leary et al., 2013; Zhang et al., 2012; Flom et al., 2025; Kocharyan et al., 2026; Barron and Wynn, 2011; O'Malley et al., 2016; Kamakura and Nadol, 2016). Under these conditions, dendritic cells may sample biomaterial-associated proteins, cellular debris, or altered self-antigens and coordinate downstream T-cell responses (Flom et al., 2025; Kocharyan et al., 2026). Such mechanisms could help explain the chronicity and complexity of cochlear implant-associated inflammation beyond macrophage-only models. This observation is particularly noteworthy in the traditional view of considering the cochlea as an immune-privileged organ with limited adaptive immune involvement (Seyyedi and Nadol, 2014). The findings here challenge this paradigm and support emerging evidence that the inner ear generates antigen-specific immune responses under pathological conditions, including after cochlear implantation (O'leary et al., 2013). The presence and activation of dendritic cells at the interface between the implant surface and surrounding stromal and immune cells imply a coordinating role in the foreign body response. Dendritic cells in many peripheral tissues shape macrophage behavior and fibrotic outcomes by modulating cytokine gradients and T cell polarization (Oggero et al., 2022; Flom et al., 2025; Wynn, 2004).

### 4.3 Microglia-like cells represent a specialized neural-associated immune compartment

The presence of microglia-like cells in discrete regions of cochlear subregions adds further complexity to the inner ear immune landscape. We use TMEM119 reporter expression (Figure 2) and P2Y12 immuno-reactivity (Figure 3) to identify cells with molecular signatures consistent with homeostatic microglia, distinct from infiltrating monocytes or macrophages (Butovsky et al., 2014; Bennett et al., 2016). These TMEM119-GFP^+^ resident phagocyte cells showed a distinct regional pattern, with the highest density in the lateral wall base, followed by the modiolus and spiral ganglion base, and minimal density in the scala tympani base (Figure 2J). Notably, TMEM119-GFP^+^ cells were also consistently observed in the spiral limbus, a region outside the four quantified compartments. This observation extends the known distribution of resident, microglia-like phagocytes in the cochlea and identifies the spiral limbus as a structure adjacent to the organ of Corti and involved in tectorial membrane attachment an additional site warranting dedicated investigation in future studies. Their presence in close association with neural elements and central auditory pathways supports the concept that the cochlea shares immunological features with the central nervous system, including resident microglial populations poised for neuroimmune surveillance (Prinz et al., 2019; Kierdorf and Prinz, 2017).

In contrast to the implant-associated increase we observed for CD11c-GFP^+^ cells, TMEM119-GFP^+^ cell density did not change significantly in any region following implantation compared to the contralateral cochlea (Figures 5U–X), despite modest, non-significant trends toward increased density in the scala tympani and spiral ganglion and decreased density in the lateral wall. Following cochlear implantation, TMEM119 expression persisted in cells adjacent to the implant site, indicating either activation of resident microglia-like cells or phenotypic adaptation in response to injury.

To validate TMEM119-GFP^+^ cells as a resident, microglia-like phagocyte population in the cochlea, we assessed co-expression of the canonical microglial marker P2Y12. TMEM119-GFP^+^ cells consistently co-expressed P2Y12 in the stria vascularis, spiral ligament, spiral limbus, and modiolus (Figure 3), supporting their identity as resident, microglia-like mononuclear phagocytes. Notably, P2Y12 immunoreactivity was restricted to these TMEM119-GFP^+^ cells and was not observed on spiral ganglion neuron cell bodies, reinforcing that the P2Y12 signal detected here reflects a phagocyte population rather than a non-specific or neuronal source.

### 4.4 Divergent spatial patterns between the two populations suggest distinct functional niches

The differing regional distributions of CD11c-GFP^+^ and TMEM119-GFP^+^ cells, the former enriched in the spiral ganglion and the latter in the lateral wall, suggest that these two phagocyte populations occupy at least partially distinct anatomical niches and may serve complementary rather than redundant surveillance functions. This regional divergence is consistent with emerging models of tissue-resident macrophage heterogeneity in other organs, where local microenvironmental cues shape phagocyte identity, density, and function on a region-by-region basis rather than producing a uniform population throughout the tissue. This divergence is raising important questions about lineage boundaries and functional plasticity among mononuclear phagocytes. Here, Figures 4, 5 support a model in which cochlear implantation preferentially engages a recruited or locally activated CD11c-GFP^+^ phagocyte population while leaving the tissue-resident TMEM119-GFP^+^ population comparatively undisturbed. This dissociation parallels findings in the central nervous system, where resident microglia and infiltrating monocyte-derived macrophages respond differently to injury, with resident cells often maintaining baseline surveillance functions while recruited cells drive the acute inflammatory response (Yamasaki et al., 2014; London et al., 2013). Although TMEM119 and P2Y12 are regarded as microglia-specific markers in the brain, emerging evidence advocates context-dependent expression in peripheral or border-associated macrophages under certain conditions (Goldmann et al., 2016; Van Hove et al., 2019). We used multiple markers to underscore the necessity of combinatorial approaches to accurately define cochlear immune cell identities, particularly in disease or injury states where phenotypic convergence may occur.

### 4.5 Translational implications for cochlear implantation

From a translational viewpoint, these findings carry important implications for cochlear implant design and post-implantation management. The prominent role of antigen-presenting cells suggests that adaptive immune modulation could represent a novel therapeutic approach to mitigate chronic inflammation and fibrosis. Local delivery of immunomodulatory agents targeting dendritic cell activation, MHCII expression, or T cell recruitment may help preserve cochlear architecture and improve long-term implant performance. In a similar way, strategies targeting modulation of purinergic pathways could selectively dampen a harmful neuroinflammatory response while preserving protective immune functions.

This study also highlights the cochlea as a valuable model for investigating neuroimmune interactions at the interface of biomaterials and sensory neurons. In contrast to the brain, the cochlea enables precise spatial mapping of immune cells relative to implanted devices, neural structures, and vascular compartments. This accessibility provides unique opportunities to dissect cellular and molecular mechanisms underlying foreign body responses in neural tissues, with relevance extending beyond auditory prostheses to other implantable neurotechnologies (Salatino et al., 2017).

### 4.6 Study limitations and future directions

There are important limitations when interpreting these findings. First, although reporter mice and immunostaining provide strong evidence for the presence of a distinct mononuclear phagocyte population, this study did not directly assess antigen presentation, cytokine production, or T cell activation within the cochlea. Future studies, including single-cell transcriptomics, fate mapping, and functional perturbations, are essential to delineate lineage relationships and causal roles in the foreign body response (Masuda et al., 2020; Hammond et al., 2019). Second; this study was performed at a single time point post-implantation, limiting insight into the temporal dynamics of immune cell recruitment and phenotypic transitions. Longitudinal studies capturing the acute, subacute, and chronic phases of implantation will further refine our understanding of the cochlear immune response. Third, we could not differentiate tissue macrophages from monocyte-derived macrophages. CCR2 is a marker critical for the recruitment of monocytes into tissues (Tsou et al., 2007) it can be used to identify monocyte-derived macrophages in the cochlea. Fourth, although we identified a population of dendritic cells in the cochlea, we did not further subclassify them. Three major classes of dendritic cells have been described (Collin and Bigley, 2018). Future studies will be required to subclassify the cochlear dendritic cells described here. A fifth limitation of our study is that these reporter mice are on the C57/B6 background. Although they have normal hearing at the time used in the study, these mice develop early-onset age-related hair cell and hearing loss (Jurga et al., 2020; Ison et al., 2007). Thus, the activation of the immune response, e.g., infiltration of mononuclear phagocytic cells, and expression of inducible markers (e.g., MHCII) might be associated with this cochlear pathology. Also, developmental changes might be an important factor determining the behavior of cochlear mononuclear phagocytes. Therefore, we used relatively young mice (10–12 weeks). A study of mice carrying the two transgenic reporters (TMEM119, and CD11c) on a background with relatively stable hearing (e.g., CBA/J) could resolve these issues. Sixth, because of the modest per-group sample sizes (*n* = 3–4 mice), we did not test for sex-specific differences and instead pooled data from male and female mice; larger, adequately powered studies will be required to examine potential sex-based differences in cochlear mononuclear phagocyte populations.

## 5 Conclusion

Overall, our data suggest that the mononuclear phagocytic system in the cochlea comprises various cell types with different regional distributions and responses to cochlear implantation. In broad terms, dendritic cells appear to function as early antigen-presenting responders poised to engage adaptive immunity, while microglia-like cells likely contribute to neuroimmune signaling within cochlear neural compartments. These cells have distinctive spatial organizations in both uninjured healthy and implanted cochleae. A subset of cochlear mononuclear phagocytes (i.e., dendritic cells) expresses MHCII, a marker of antigen-presenting cells, suggesting that MHCII-mediated adaptive immunity functions in the cochlea. These findings help define the immunological landscape of the inner ear and provide a framework for developing immune-informed strategies to improve the safety and efficacy of cochlear implants. Follow-up studies, both fundamental and translational, can delve further into the functional significance of these cells.

## Data Availability

The raw data supporting the conclusions of this article will be made available by the authors, without undue reservation.

## Footnotes

1
https://umgear.org/

## Supplementary material

SUPPLEMENTARY FIGURE 1 Validation of the CD11c-GFP reporter using Diphtheria toxin mediated controls. (A&C) PBS treated gut tissue showing CD11c-GFP signal (A, green), merged image with DAPI (C, blue), confirming specificity and tissue architecture. Diphtheria toxin mediated depletion (B & D), demonstrating substantial depletion of CD11c-GFP signal (B), with DAPI labeling to confirm tissue architecture (D). Scale bar = 50 µm.

SUPPLEMENTARY FIGURE 2 Validation of TMEM119-GFP reporter by co-localization with microglial markers in the brain. (A–C) TMEM119-GFP signal (A, green), anti-P2Y12 immunolabeling (B, magenta), and (C) merged image of TMEM119-GFP (green) and anti-P2Y12 (magenta) showing co-localization in the same cells. (D-F) Same fields with DAPI (blue), (D) DAPI with TMEM119-GFP, (E) DAPI with anti-P2Y12, and (F) DAPI with TMEM119-GFP and anti-P2Y12. Scale bar=20µm.

SUPPLEMENTARY FIGURE 3 Distribution of CD11c-GFP^+^ and MHCII^+^ cells at the implant interface in an implanted cochlea. (A–C) Showing CD11c-GFP signal (A, green) and anti-MHCII immunolabeling (B, red) within the scala tympani at implant interface, with a merged image (C). (D–F) with DAPI to confirm tissue architecture. Scale bar= 50 µm. Asterisks (*) indicate the implant track.
